# Protein Albumin Manipulation and Electrical Quantification of Molecular Dielectrophoresis Responses for Biomedical Applications

**DOI:** 10.3390/mi13081308

**Published:** 2022-08-13

**Authors:** Nur Shahira Abdul Nasir, Revathy Deivasigamani, M. F. Mohd Razip Wee, Azrul Azlan Hamzah, Mohd Hazani Mat Zaid, Muhammad Khairulanwar Abdul Rahim, Aminuddin Ahmad Kayani, Abdullah Abdulhameed, Muhamad Ramdzan Buyong

**Affiliations:** 1 Institute of Microengineering & Nanoelectronics (IMEN), Universiti Kebangsaan Malaysia (UKM), Bangi 43600, Selangor, Malaysia; 2 Functional Materials and Microsystems Research Group and the Micro Nano Research Facility, School of Engineering, RMIT University, Melbourne, VIC 3001, Australia; 3 Department of Electronics & Communication Engineering, Faculty of Engineering & Petroleum, Hadhramout University, Al-Mukalla 50512, Hadhramout, Yemen

**Keywords:** dielectrophoresis, electrical quantification, protein, manipulation

## Abstract

Research relating to dielectrophoresis (DEP) has been progressing rapidly through time as it is a strong and controllable technique for manipulation, separation, preconcentration, and partitioning of protein. Extensive studies have been carried out on protein DEP, especially on Bovine Serum Albumin (BSA). However, these studies involve the usage of dye and fluorescent probes to observe DEP responses as the physical properties of protein albumin molecular structure are translucent. The use of dye and the fluorescent probe could later affect the protein’s physiology. In this article, we review three methods of electrical quantification of DEP responses: electrochemical impedance spectroscopy (EIS), cyclic voltammetry (CV) and capacitance measurement for protein BSA DEP manipulation. The correlation of these methods with DEP responses is further discussed. Based on the observations on capacitance measurement, it can be deduced that the electrical quantifying method is reliable for identifying DEP responses. Further, the possibility of manipulating the protein and electrically quantifying DEP responses while retaining the original physiology of the protein and without the usage of dye or fluorescent probe is discussed.

## 1. Protein as the Basic Component of Life

Protein dielectrophoresis (DEP) has been a topic of interest since almost 30 years ago [1]. Protein DEP has good prospects, providing advantages such as higher output and efficiency, uncomplicated system setup, and a lower probability of biosample contamination [2]. Protein DEP has a promising future to overcome the drawbacks of conventional protein analysis methods. As these drawbacks are resolved, protein DEP methods can be developed into fully functional bio-analytical devices, which could offer advancements in medical device technology.

Emerging diseases have prompted extensive medical research to extend life expectancy. Interdisciplinary studies have been rapidly progressing to overcome the threat of terminal diseases. Research and development are evolving rapidly to meet biomedical healthcare demands and overcome obstacles such as high cost, complicated preparation methods, minimizing side effects and capital-intensive medical procedures. 

Proteins, also known as polypeptides, are known to be the basic component of every living cell of all forms of living, from prokaryotes to eukaryotes, including bacteria and viruses. Their various functions include enzymes or catalytic proteins, which hold genetic information and can become a cell’s main support system (cytoskeletal) [3]. The structures of protein can be classified into 4-four groups which are primary, secondary, tertiary and quaternary. These structures are usually connected by a few non-covalent bonding such as hydrogen bonding, ionic interactions, Van der Waals forces, and hydrophobic interaction [4]. The primary structure consists of only a strand of amino acid sequence. In contrast, the secondary structure consists of a few strands of amino acid sequence, which are usually connected by a hydrogen bond. The tertiary structure is a frame of protein where few secondary structures are connected. The quaternary structure is the most complex protein structure. A few protein molecules form it joined together as a sole protein complex.

**Figure 1:**
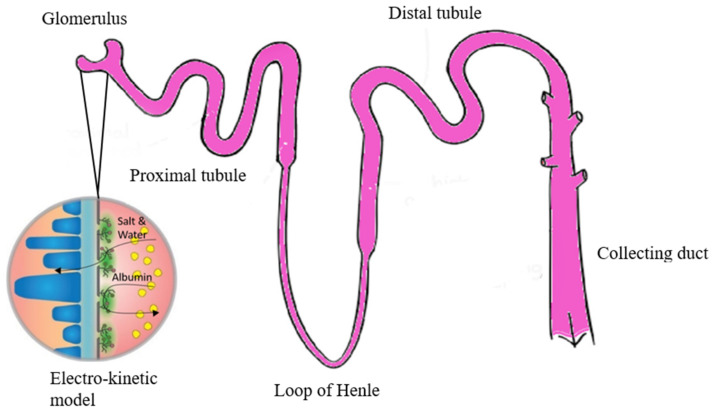
Renal physiology of the kidney.

Albumin is a specific protein that plays an important role in the body’s wellbeing. Serum albumin (S-Alb) is the largest protein component of human blood (50–60%) and is an important factor in regulating plasma volume and tissue fluid balance. S-Alb was formerly considered a biomarker of visceral protein and immunocompetence status, fundamental to nutritional assessment. Albumin is synthesized in the liver, and its main function is it helps in regulating the oncotic pressure [5]. Albumin is also known to have other multiple functions in which it will attach and transport abundant endogenous and exogenous components [6]. The main reason that albumin should be retained in the blood is its various functions. In the kidney, albumin is filtered in the glomerulus, reabsorbed in the proximal tubule, then by a loop of Henle and the distal tubule. Finally, the reabsorption process ends by the collecting duct [7]. Figure 1 below shows the renal physiology of the kidney.

However, patients with end stage renal disease (ESDR) indicated that nephrotic failure would have difficulty keeping albumin in the blood [8]. A condition called proteinuria is a nephrotic syndrome where excessive albumin is found in the urine. This indicates the failure of the kidneys. Diseases associated with albumin found in the body are listed in Table 1 below.

Human serum albumin (HSA) has shown a heart-shaped tertiary structure using X-ray crystallography. However, the shape then shifted to be ellipsoidal when dissolved in solution [16]. As for bovine serum albumin (BSA), it has been shown that BSA possesses a prolate ellipsoidal shape, proven by hydrodynamic measurements of sedimentation coefficients and rotational relaxation time. As reported, BSA is very similar to HSA in terms of chemical make-up, structure and physico-chemical properties [17]. The molecular weight of BSA is 66,700 Da [18]. Based on the equation below, the molecular weight can be converted to length in nanometer (nm) [19]. M represents the molecular weight of protein in Dalton while $R_{min}$ in nm. V is the volume occupied by a protein The length of BSA is 27 nm.

(1) $$R_{min}=0.066M^{\frac{1}{3}}$$

(2) $$R_{min}=0.066\;(66,700{)}^{\frac{1}{3}}$$

BSA can be identified through protein analysis methods. Protein analysis methods are carried out to study proteins’ function and structure [20] thoroughly. It is important to study these characters of protein to be able to understand their interaction. Precise protein detection is vital. Some of the protein analysis methods which have been used widely are enzyme-linked immunosorbent assay (ELISA), western blotting [21], mass spectrometry [22] and gel electrophoresis [23]. However, these conventional protein analysis methods have a few drawbacks listed in Table 2 below. Some protein analysis methods can be modified to ensure a more precise result.

**Figure 2:**
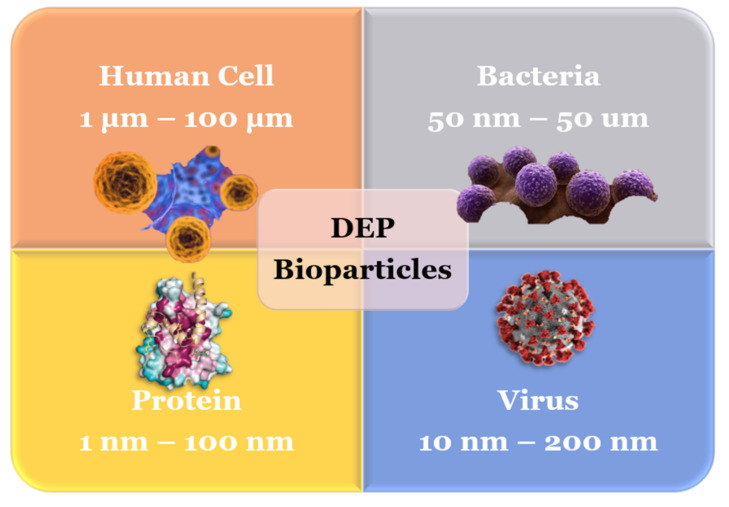
Bioparticles that have been studied with DEP [43].

In order to overcome these weaknesses, the concept of electrical properties of biomolecules can be implemented to ensure precise segregation and manipulation of protein. The interconnection of bioparticles involving cells, viruses, proteins and bacteria with the electric field has been studied because of their possible importance in the biomedical sector [35]. Dielectrophoresis (DEP) is defined as the movement due to the nonuniform electric field exerted on the particle [36]. DEP is one of the best methods of protein analysis because it offers a contactless, label-free method, with high precision [37,38,39,40]. DEP has been proven to manipulate and actuate bioparticles of various sizes [41,42]. Figure 2 below shows the several bioparticles associated with DEP that have been studied extensively throughout the years.

Protein DEP has been studied immensely, but the concluding remarks always end with another question as protein DEP theory is different from the standard DEP theory used for biomacromolecules. In this article, we will explain more about the correlation of DEP for effective protein manipulation and some of the electrical quantification techniques of the DEP response, where no fluorescent labeling of the protein is required.

## 2. Theory of DEP

DEP theory was discovered by Herbert Pohl in the 1950s when the sample used was on liquidated particles [44,45]. In his paper, he refers to the DEP response as ‘dielectro-precipitation’, which is when the polarizability of the liquidated particles is bigger than the solvent, causing the concentrated suspension to be more susceptible to collisions or congelation. DEP theory has been further studied in terms of the manipulation of bioparticles. In a recent study, research on DEP is implemented in dialyzers of kidney hemodialysis [46,47].

**Figure 3:**
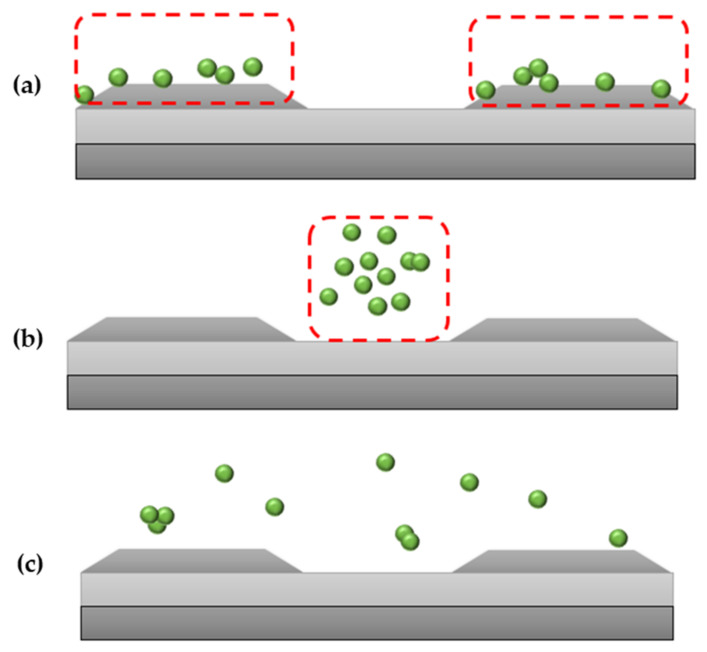
(**a**) pDEP or positive-DEP where particles are seen to be attracted to the edge of the electrodes. Particles move to the area with a higher field gradient, (**b**) nDEP or negative-DEP, where particles are seen repelled towards the center of the electrodes. Particles move away from the area with a high field gradient, (**c**) $f_{xo}$ also known as crossover frequency, where particles can be seen dispersed around the electrodes.

DEP allows concentrating, transferring, capturing, refinement, and enhancement of biological or clinical samples [48,49]. There are two types of DEP responses that highly rely on the conductivity and permittivity of the suspending medium and the bioparticles [50]. pDEP, also known as positive-DEP, is when the particles exerted with force are seen to be attracted to the edge of the electrodes (regions with high electric field). nDEP, also known as negative-DEP, is when the particles exerted with force are seen to be repelled towards the center of the electrodes (regions with low electric field). These DEP responses, as shown in Figure 3a–c, are expected of protein when being manipulated with DEP [51]. 

In DEP, the particles subjected to manipulation can be applied with either alternating current (AC) or direct current (DC) nonuniform electric field [52]. However, ideally, AC is more suitable for DEP because DC tends to give rise to Faradaic reaction due to electrode-electrolyte interaction causing the formation of bubbles and other undesired responses [53]. The time-averaged DEP force ($F_{DEP}$) exerted on spherical particles with radius, *r* can be calculated as in the equations below [54,55,56,57].

(3) FDEP=2πε0εmr3Re (CMf) ∇|E2|

(4) $$\mathrm{CM}f=\frac{\varepsilon_{p}^{*}\;-\;\varepsilon_{m}^{*}}{\varepsilon_{p}^{*}\;+\;2\varepsilon_{m}^{*}}$$

(5) $$\varepsilon^{*\;}=\varepsilon -j\;\frac{\sigma }{\omega }$$

$\varepsilon_{p}$ represents the permittivity of the particle while $\varepsilon_{m}$ is the permittivity of the medium. $\varepsilon^{*}$ is the complex permittivity which can be calculated in Equation (6). *σ* is the conductivity, and *ω* is the angular frequency. $\varepsilon_{0}$ is the vacuum permittivity, where the value is 8.854 × 10^−12^ F/m [48]. CM*f* is the Clausius-Mossotti factor, also known as the Maxwell-Wagner factor. CM*f* contains two parts which are the real part and the imaginary part. The real part of CM*f* dictates the force in the direction of the highest or lowest point of an electric field. pDEP and nDEP responses can be determined by CM*f*. 

The CM*f* is at its highest during pDEP and lowest during nDEP [58]. When the particle is seen to be more polarizable than the suspending medium (Re(CM*f*) > 0), the DEP response will be pDEP. When the particle is seen to be less polarizable than the suspending medium (Re(CM*f*) < 0), the DEP response will be nDEP [59]. Protein albumin is known to be ellipsoidal [60]. The equation to calculate $F_{DEP}$ exerted on ellipsoidal particle can be calculated as below:

(6) FDEP=2πaextbextcextεm Re (CMf) ∇ |E2|

where a stands for dimension on the *x*-axis, b is on the *y*-axis, while *c* is on the *z*-axis [61]. CM*f* can be calculated as the equation below:

(7) $$\mathrm{CM}f=\;\frac{C\;M_{x\;}f+C\;M_{y}\;f+C\;M_{z}f}{3}$$

(8) $$CM_{\alpha }f=\frac{1}{3}\frac{\varepsilon_{p}^{*}-\varepsilon_{m}^{*}}{(\varepsilon_{p}^{*}-\varepsilon_{m}^{*})A_{\alpha }+\varepsilon_{m}^{*}}$$

*α* stands for *x*,*y* or the *z*-axis while $A_{\alpha }$ is the depolarization factor. Based on the paper written by [62], proteins have a permanent dipole moment due to their interaction with water hydration forming hydration sheath and the total charges interaction within the protein cavity [63]. This is different from other biomacromolecules such as cells, bacteria, virions and up to vesicles that possess an induced moment. A dipole moment can be defined as the separation of charge within a region. 

For a normal induced dipole moment model of DEP exerted on a spherical particle, the real part of the CM*f* value ranges from 1.0 > CM*f* > −0.5 [64]. According to Pethig, the dipole moment affects DEP response [62]. Equation (10) below is described to explain the $F_{DEP}$ of a molecular structure of spherical *E. coli* rRNA, which is a form of protein:

(9) $$F_{DEP}=[(\mu_{p}+\mu_{ind})\;\nabla ]E_{o}=\mu_{p}\nabla \;E_{o}+\frac{1}{2}\alpha_{T}v\nabla E_{o}^{2}$$

$\mu_{p}$ is the permanent dipole moment while $\mu_{ind}$ is the induced dipole moment. A is the polarizability, and *v* is the volume. The symbol ∇ $E_{o}$ could contribute to a pDEP response. ∇ $E_{o}^{2}$ plays an important role in an nDEP response. For manipulation of protein using DEP, the DEP force is exerted by the surrounding suspending medium of the protein. However, this equation is not applicable for globular protein. This shows that the equation for calculating DEP force is different for calculations of protein from the standard force of DEP equation.

In 2022, Pethig further developed an equation for protein DEP with a permanent dipole moment [63], where the DEP response of protein DEP is not relevant using the common DEP theory. As the conventional equation of DEP force heavily relies on the shape and size of the particles with induced dipole moment, the case is different with protein. This is because the aspect that could affect the DEP force includes the dielectric dispersions caused by the protein’s electrical double-layer, the permanent dipole characteristics, the hydration shell and the interaction of the protein with water, respectively. 

**Figure 4:**
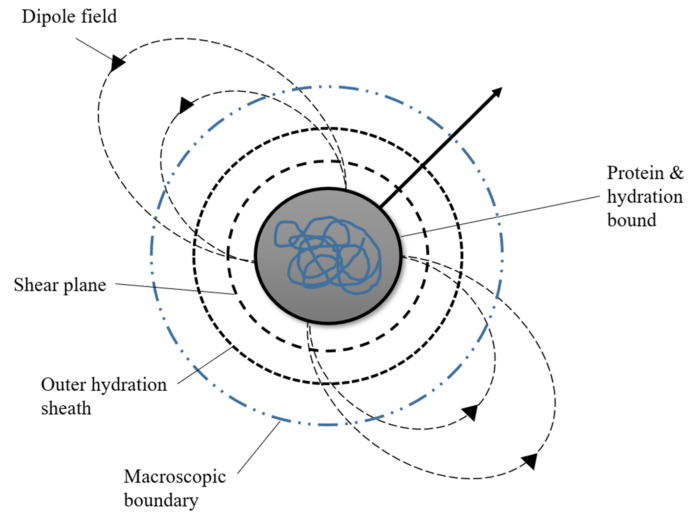
‘Russian doll’ model of a protein. The protein is surrounded by a permanent dipole moment. The protein fills the most internal cavity which is strongly attached to water molecules.

The protein with the permanent dipole moment is the water molecule’s central orbit, as shown in Figure 4. This will be further discussed in the protein ‘Russian Doll’ theory proposed by Holzel and Pethig [64].

The dipole field of protein projects outwards, surpassing the macroscopic boundary. According to Sayedi & Matyushov [65], proteins have a large amount of surface charge for their solubility in suspending medium. The negative and positive charges recompense to produce a net negative charge at their physiological state. However, the protein which is cytochrome-c studied by [65] is positively charged. The proteins’ irregular shape and non-balanced overall charge cause a huge dipole moment. The discussion on factors affecting DEP will be elaborated.

Several electrokinetics cases could highly affect DEP. As stated by [1], some of the factors are electroosmosis, thermal heat force (Joule Heating), electrokinetic force, hydrodynamic force, and Brownian motion. By minimizing the effect of these interferences, DEP force could be maximized. Electroosmosis is the fluid motion of the particles after being applied with frequency due to the viscosity of the medium [66]. The other case that could highly affect DEP is Joule Heating, also known as electrothermal motion, which is the movement in mediums due to the temperature gradient when electric current flows through an electrical conductor [67,68]. A study was conducted on the factors such as the diameter and distance of the electrode and channel height affecting Joule Heating [69].

Brownian motion is defined as the random movement of particles suspended in a medium [70]. This could also affect DEP response [71]. This phenomenon that needs to be overcome by DEP is the dispersive force linked to Brownian motion [63]. As the size of particles gets smaller to the nanometric scale, the DEP force becomes weaker, and it tends to be subjected to increasingly Brownian motion [72]. Thus, it is harder to manipulate using DEP as the size gets smaller.

**Figure 5:**
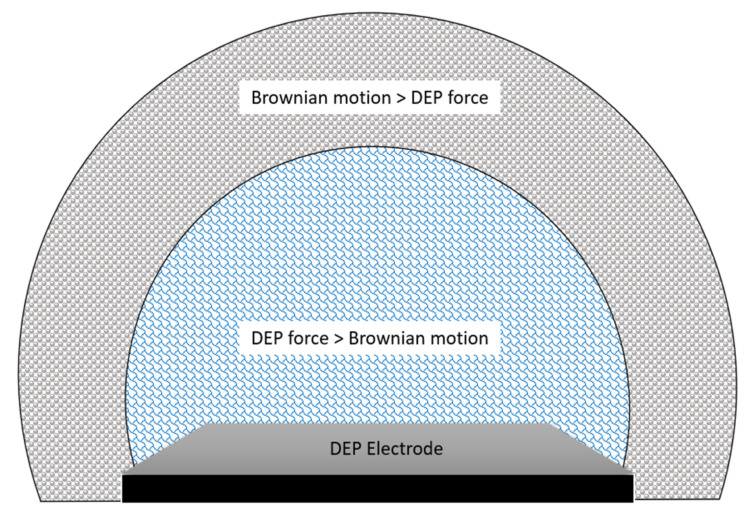
DEP force versus Brownian motion in terms of area of force concentration.

The general phenomenon of Brownian motion in DEP is well-explained in the Fokker-Planck (or Smoluchowski) equation [73]. This equation is also known as the diffusion-advection equation. This equation has considered both pressure-driven and electrohydrodynamic flows for DEP simulation. The Brownian motion becomes weaker as it extends outwards the electrode region as the DEP force is strongest at the tip of the electrodes. DEP force can be neglected as it gets further from the electrodes, and the force of liquid diffusion takes over. The distribution of the Brownian motion compared with DEP force is visualized in Figure 5 below.

## 3. DEP Studies on Protein Albumin

BSA is the most studied protein for DEP characterization. Basically, two types of electrodes configuration are being studied for BSA. The first type is insulator-based DEP (iDEP) and the other type is electrode-based DEP (eDEP). The definition of two types of DEP associated with studies with sample BSA is listed below in Table 3.

The results obtained from albumin protein DEP studies and respected frequency are listed in Table 4 and Table 5 below:

Most of the studies of BSA were observed for pDEP but only two studies with nDEP of iDEP were reported [83,84]. According to Nakano et al. [81], nDEP results because the iDEP needs to overcome the problem of controlling the protein aggregates. Research has also been carried out to study the electrical properties of BSA by observing the impedance of BSA as BSA accumulates onto the electrodes [90].

The principle of eDEP is well-established as DEP was first found using the eDEP principle. iDEP was later developed through time [91]. As Benhal et al., (2020) stated, Joule heating and disintegration of electrodes are more likely to happen in eDEP as a higher gradient is being created. This could subsequently contaminate the biological sample [74]. According to Pethig, the usage of eDEP is more relevant for smaller molecules such as proteins, virions, vesicles and exosomes where high values of (*E*·∇)*E* are acquired, where *E* represents the electric field [79]. This is because the DEP manipulation of the targeted molecules will be based on the difference in dielectric properties of the membrane or ‘sheath’ and the charges of the inner cavity of the targeted particles.

Research has also been carried out by Washizu et al., (1994) on developing a protocol for identifying pDEP for protein for eDEP. The protocol describes that the first step is to identify the purity of the protein monomers using a gel chromatography method. Then, the concentration of the protein should be below 0.1 µg/mL. As for the conductivity of the suspending medium, the value should be below 1 mS/m [92]. This protocol has been widely used ever since for eDEP. 

## 4. Theories of Electrical Quantification Techniques for DEP Protein

Protein BSA is known for its translucent properties and small size [93]. Thus, making them unable to be visualized under the microscope. There have been studies conducted on BSA in which the protein binds with fluorescent probes or dye to make it visible where the data is tabulated qualitatively. Some chemicals that can be used to fluorescently label BSA are fluorescein isothiocyanate [94], 5-(4,6-dichloro-s-triazin-2-ylamino) fluorescein hydrochloride (DTAF), Rhodamine B isothiocyanate (RITC) and Lucifer yellow VS (LY) [95]. However, these chemicals might affect the dielectric properties of BSA. This will eventually cause difficulties if these principles are to be implemented into biomedical devices (such as dialyzers in kidney hemodialysis treatment) in which BSA samples are obtained without the probe and expected to be retained in such way. 

**Figure 6:**
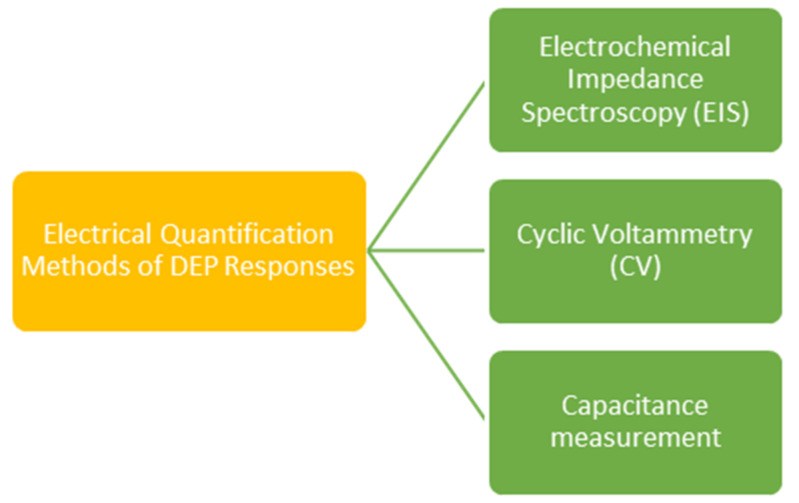
Electrical quantification methods for identifying DEP responses.

This is why it is important to find another technique to ‘visualize’ the protein or simply do a quantitative analysis of the protein’s DEP responses without the protein being visible. Some known techniques are listed in Figure 6: electrochemical impedance spectroscopy (EIS), cyclic voltammetry (CV) and capacitance measurement. These are discussed in this section and explained further.

### 4.1. Electrochemical Impedance Spectroscopy (EIS)

**Figure 7:**
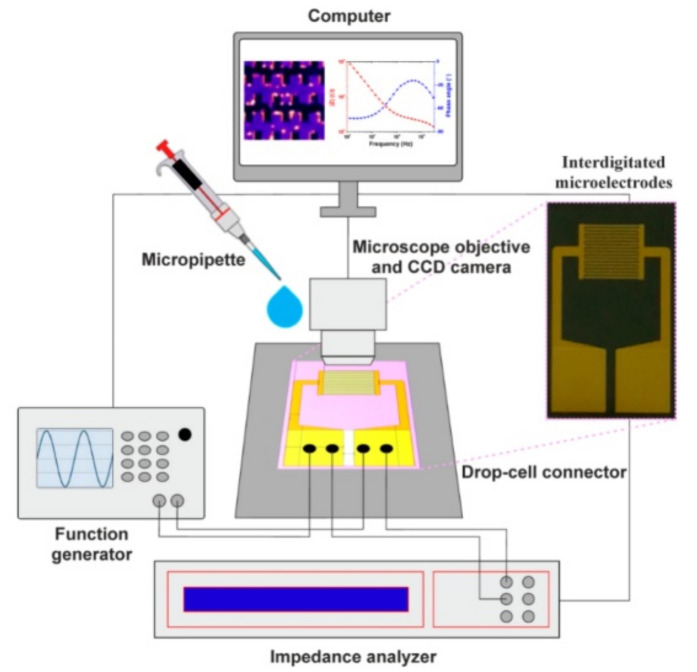
Experimental setup of DEP manipulation and EIS method. The EIS measurement results are usually in the form of Nyquist or Bode plots. Reprinted with permission from Ref. [97], ©MDPI 2021.

EIS, also known as electrochemical impedance spectroscopy, can be defined as the measurement of the impedance of a setup that depends on AC potentials frequency. This technique heavily relies on the movement of the charges across the configuration of the electrodes in contact with the analyte [96]. For the measurement of EIS, the impedance of the electrodes is plotted versus frequency [97]. The definition of impedance is the effective resistance in an electrical circuit. While resistance is a concept applicable to DC, impedance is a concept that is applicable to AC. In short, impedance means the effective resistance when current flows through a circuit consisting of resistors, capacitance and inductors. Figure 7 shows the schematic arrangement of apparatus for EIS measurement. 

Research on the manipulation of cancerous cells using DEP recorded in 2021 has shown the connection of the impedance characteristics based on DEP parameters with the physical characteristics of the cancerous cells. The result suggested that the unique electrical properties of the biomolecules can be a future perspective on differentiating cancerous cells from normal cells [97]. With this implementation, this concept can also be implemented for protein manipulation for artificial kidney applications. 

Most EIS measurement integrated with DEP is being applied with eDEP [98,99]. However, there has been an issue concerning linking EIS with eDEP. The design of the electrodes in a microfluidic device should envelop the whole height of the microfluidic channel, or else this could cause an error in EIS measurement. EIS is famous for detecting DEP response as they are a label-free technique. There have been studies in which EIS has been used to uncover the DEP properties of DNA by measuring the capacitance between the electrodes [100]. 

There are some disadvantages of EIS. One of them is EIS is a cost-consuming method. Not to mention that EIS data analysis can be protracted and complicated. As a result, it is highly reactive to electrode contamination [101]. Other than that, EIS characterization is highly reliable on different rates of frequencies. The impedance measurement can take up to a few hours and relies on the frequencies and the potential being applied. Regrettably, within a longer time frame, the analysis system can change. EIS’s high dependency on the system is the major drawback of the EIS method [102].

### 4.2. Cyclic Voltammetry (CV)

**Figure 8:**
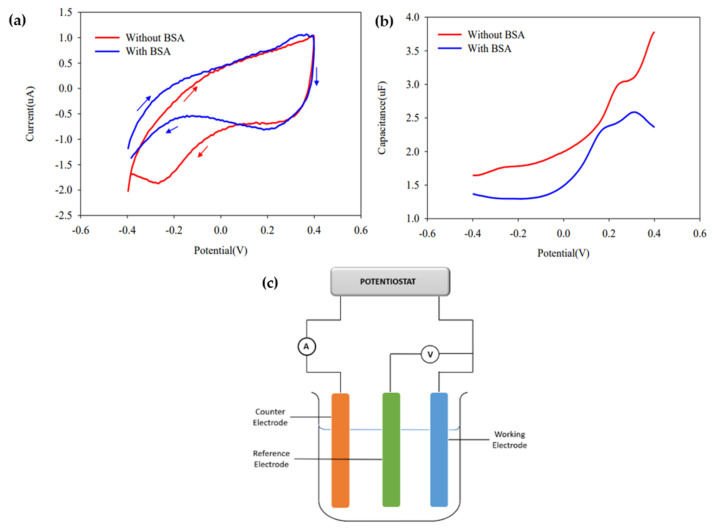
(**a**) The voltammogram graph, (**b**) the curve measurement of capacitance. The potentiostat can do both measurements (**c**) Three types of electrodes dipped into the analyte for CV. Adapted with permission from Ref. [102], Madduri 2012.

Another method that has not been studied extensively is cyclic voltammetry (CV). CV is also a method that measures the current that flows through an electrochemical cell with the condition of voltage being in abundance following the Nernst equation. In a CV process, the electrode is immersed in an analyte with voltage applied through the electrode. The system response is then recorded in a voltammogram in which plots are drawn to show current versus potential. The electrodes involved in a CV system are counter, working and reference electrodes, as shown in Figure 8 below. 

### 4.3. Capacitance Measurement

The last method we propose for electrically quantifying DEP responses is capacitance measurement. Capacitance can be defined as the capability of a conductor to store electrical charge. Some of the known equipment used for capacitance measurement of DEP responses are the LCR Metre [103]. For capacitance measurement of DEP responses, the reading depends on the concentration of the biosample in the area between DEP microelectrodes during the sensing time [104]. In general, the capacitance value depends on a few key principles: the dielectric properties, the distance between the electrodes and the surface area of the electrodes. The equation for calculating the capacitance of a capacitor can be calculated in terms of the area and dielectric properties. The equation is expressed in Equation (11) below:

(10) $$C=f\;(d,\;A,\;\varepsilon_{r})$$

In this equation, $\varepsilon_{r}$ is the relative static permittivity, also known as the substance’s dielectric constant located between the electrodes. *A* is the surface area of the electrodes, while *d* is the distance between the electrodes [105]. Each key principle plays an important role in the calculation of capacitance. The change in capacitance can be used to detect the bioparticles in between the electrodes.

## 5. Correlating the Principles of Electrical Quantification Methods to DEP

The electrical quantification method options can be used to quantify the DEP responses without the use of dye or fluorescent probe, thereby retaining the original physiology of the protein albumin. Further analysis of the principle for both mechanisms makes both techniques suitable to be linked with DEP. Table 6 below discusses more on the concept of principles of EIS, CV and capacitance measurement. 

**Figure 9:**
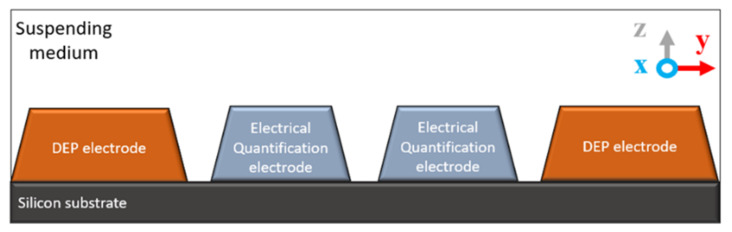
Integrated DEP & electrical quantification electrodes.

The ideal geometry of electrode configuration that contains both DEP and electrical quantification electrodes is shown below in Figure 9. The fabrication of the electrodes begins by depositing a layer of 11.5 K Å thick silicon dioxide onto the top surface of a silicon wafer substrate. The silicon dioxide layer on the top of the wafer acts as an insulator between the silicon substrate and before the metal deposition layer. Secondly, an additional layer thickness of 600 Å/300 Å of Ti/TiN acts as a barrier and adhesion layer before a third additional layer thickness of 40 K Å of aluminum (Al). The Al layer on the wafer is patterned using the standard photo-lithography technique, consisted photoresist coating, exposure, and development. Then the metal etches, and plasma resists the strip [108]. 

**Figure 10:**
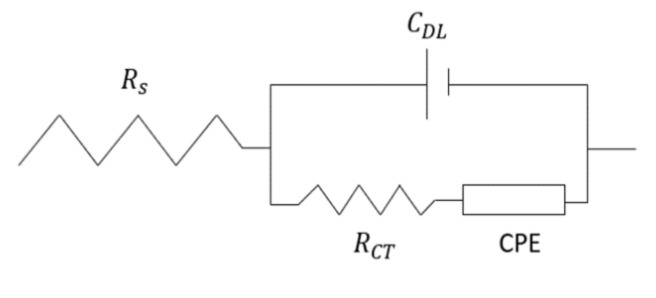
Randles circuit model.

Theoretically, the electrical impedance feature of integrated DEP and EIS electrodes can be further explained in line with the Randles circuit model, as illustrated in Figure 10 below.

In an integrated DEP and EIS electrode system, the suspending medium’s resistance is represented by the symbol, $R_{S}$ which is in a series circuit with a parallel grouping of double layer capacitance, $C_{DL}$ which constitutes the charge transfer between the electrode and the suspending medium while $R_{CT}$ and a constant phase element CPE describes the charge transfer reaction that passes through three different elements in the DEP and EIS electrode system. These are the suspending medium, the electrodes and the sample protein albumin [109,110,111]. The $R_{CT}$ and CPE values are highly affected by the physical characteristics (e.g., the dielectric properties) of the sample protein. The biological protein sample that is being used in DEP experiments does not constitute the capacitance of an ideal capacitor as they have irregular shapes and surface roughness, and thus $Z_{\mathrm{CPE}}$ = $\frac{1}{Q\;{\left(j\omega \right)}^{n}}$, where Q is the magnitude of $Z_{\mathrm{CPE}}$ [97,112]. With all these terms and conditions into consideration, the total impedance of the integrated DEP and EIS electrode system can be calculated with the equation below:

(11) $$Z=R_{S}+\frac{Z_{DL}\left(R_{CT}+Z_{\mathrm{CPE}}\right)\;}{Z_{DL}+R_{CT}+Z_{\mathrm{CPE}}}$$

(12) $$Z_{DL}=\frac{1}{j\omega C_{DL}}$$

where ω is the angular frequency and the value of *n* is constant (0 ≤ *n* ≤ 1). By calculating the Z value based on different DEP responses from the above equations, we can deduce the DEP responses. For nDEP response, the particles are concentrated in the centre of the integrated electrodes, increasing the capacitance measurement. As the capacitance increases, the Z value calculated will increase. With the Z value obtained, we can hypothesize that the DEP response that occurs is nDEP. In a vice versa response, where particles are being repelled away from the center and attached to the DEP electrodes, the capacitance decreases. Thus, the Z value calculated will decrease. From the Z value obtained, we can deduce that the DEP response that occurred is pDEP. 

A similar concept can be applied for translating voltammogram results into a DEP response. From the peak current, which heavily relies on the adsorption of the particles onto the surface of the electrode [113], we can deduce the DEP reaction that occurs. The principle of CV for DEP response quantification is also based on the weight of the load on the ROI (Region-of-Interest). This can be identified by using samples from higher concentrations to lower concentrations. The reading of the voltammogram is based on the peak current obtained. The peak current is highly dependent on the adsorption of the sample towards the surface of the electrodes. As the samples’ concentration increases, the samples’ adsorption will also increase. This will cause the peak current of the voltammogram decreases. By studying the voltammogram obtained, we can predict the DEP response from the manipulation process. The correlation between DEP responses, adsorption of sample onto the electrodes, and the peak current of voltammogram is simplified in Table 7 below.

In conclusion, the concept of these techniques relies on the weight load located in the different parts of ROI; as the weight load is concentrated on the electrodes’ edge (pDEP response), the capacitance increases. In a vice versa reaction, the capacitance measurement decreases as the weight load is concentrated at the center area of the electrodes (nDEP response). 

**Figure 11:**
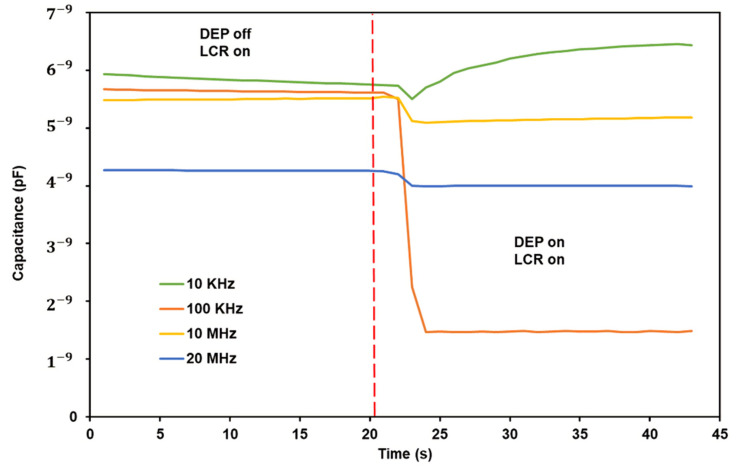
Capacitance measurement of 0.05 µm polystyrene beads and deionized water as the suspending medium.

We have conducted a preliminary experiment regarding correlating capacitance measurement to molecular DEP responses. In this experiment, the instrument used was an LCR Metre (LCR-6000 Precision LCR Meter-GW Instek). For the quantification of the DEP response, the capacitance measurement was 60 s. During this 60 s period, the first 20 s were without DEP force, and the last 20 s were with DEP force. The frequencies chosen for DEP parameters were 10 kHz, 100 kHz, 10 MHz, and 20 MHz with 10 Vpp. The movement of the polystyrene beads was observed under the microscope. All of the DEP parameters have a pDEP response on the polystyrene beads. To confirm this result, a numerical simulation using MyDEP software was carried out before the experiment. The numerical simulation result matches the simulation result from past research [47]. Figure 11 below shows the capacitance measurement obtained as a plot graph of capacitance vs. time.

From the result obtained, the graph’s peak shows that the capacitance can be detected after DEP force is applied. However, there is an inconsistent trend in the graph. Some errors and noises were able to be identified during this experiment. This experiment’s error is that the volume of the polystyrene beads sample should be consistent throughout each parameter. The volume of the sample does affect the capacitance measurement. As sample volume increases, the load weight increases. Thus, making the capacitance also increases. Each DEP parameter should be made with a new set of constant volume samples. 

From this experiment, we can conclude that capacitance measurement is one of the reliable methods to quantify DEP response electrically. This is based on the sample concentration placed in the area between the electrodes. For pDEP response, as the sample is being repelled from the area between the electrodes, the measured capacitance will decrease. As for nDEP response, the sample is concentrated in the area center of the electrodes.

## 6. Discussion

The future prospects of electrical quantification and DEP implementation are bright in the medical field, especially because no sample modification was made. The physical characteristics of some biomolecules can be very susceptible to contamination, and they may have a translucent physical, which makes them invisible under the microscope. These challenges can be overcome with the electrical quantification of DEP responses. We will further discuss the correlation of electrical quantification by making capacitance measurements based on different DEP responses. There have been past studies conducted on bacterial *Eschericia coli* [114] and DNA [103] for capacitance measurement for DEP responses. 

**Figure 12:**
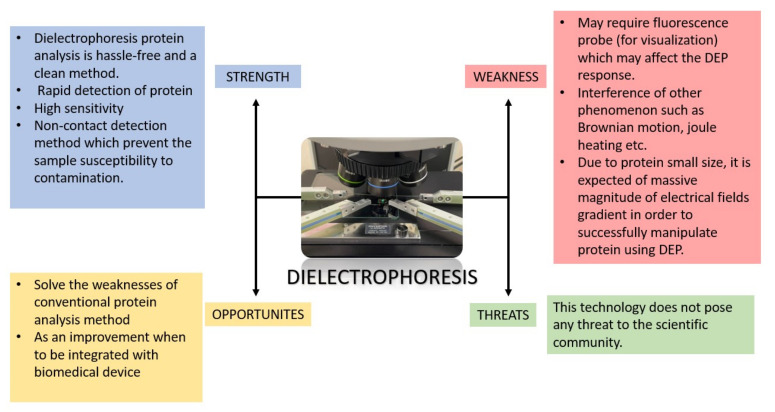
Detailed SWOT analysis of DEP.

Generally, manipulating and selective separation techniques can be categorized into two sub-classes: direct contact and non-direct contact [43]. The direct contact method has its drawbacks, especially regarding bioparticle samples, because bio-particles are more susceptible to contamination which might affect the physiology of the sample. DEP offers a label-free and precise technique to ensure the purity of the protein sample while the analysis process is being carried out. Thus, making it the most suitable method to be integrated with the protein analysis method. A ‘Strength, Weakness, Opportunity and Threat’ (SWOT) analysis was constructed based on DEP’s key principles: strengths, weaknesses, opportunities and threats. The key points are listed in Figure 12 below.

In the rest of the paper, we will discuss how the integration of DEP and quantification techniques can be implemented into biomedical devices. The biomedical device discussed in this research is the dialyzer in kidney hemodialysis treatment. With the increasing and alarming rate of diabetic patients with end stage renal disease (ESRD) throughout the world, it is vital to find a technique to ensure the effectiveness of the hemodialysis treatment. The current hemodialysis dialyzers excretion of toxic waste relies on hollow fiber membrane (HFM) [115]. The dialyzers that rely on selective separation based on pore size are highly effective for bigger molecules with the size of 15,000 Daltons and small-sized molecules with the size of 500 Daltons uremic toxins. However, the current dialyzers in hemodialysis are less efficient for molecules with a normal middle size of 500 to 15,000 Daltons [116].

Research has been written that water contamination is one of the hemodialysis treatment’s weaknesses due to the biofilm on the filter membrane. To make things much worse, the bacteria found on the haemodialysis filter are Gram-negative, making them harmful and pathogenic to humans [117]. Some Gram-negative bacteria species found in the hemodialysis filters are *Escherichia coli*, *Enterobacter* spp., *Klebsiella* spp. and *Pseudomonas aeruginosa* [118]. DEP guarantees an accurate selective manipulation of targeted particles while preventing contamination because of its non-contact characteristic [119]. 

**Figure 13:**
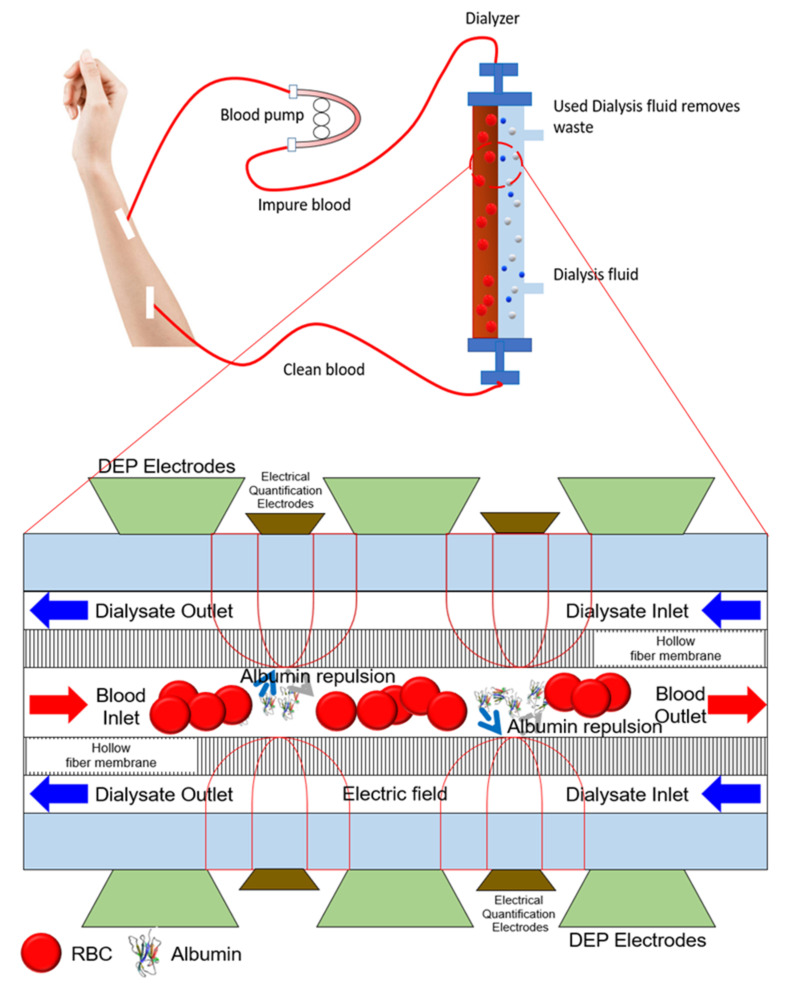
Dialyzers of hemodialysis to be integrated with DEP manipulation and quantification technique. This is one of the probable future works yet to be discovered.

Past research has proved that DEP has been explored thoroughly to improve the health status of patients with renal problems [120,121]. The principles of DEP can be used to maneuver the movement of targeted particles or proteins to be retained or expelled out of the blood. Since some of the targeted particles or proteins may be translucent in character, quantifying the movement of the targeted particles or proteins will use the chosen quantification technique. Since this paper is discussing albumin selective manipulation, the aim is for albumin to be retained in the blood. The electric field generated by the electrode will induce an nDEP response causing the albumin to be preserved in the blood. A diagram conjecture of the integration of both DEP and the selected quantification technique is shown below in Figure 13.

## 7. Conclusions

Further protein analysis with the implementation of both DEP manipulation and electrical quantification method offers a bright future. The implementation of DEP and the non-visible quantification method is not only suitable for only protein but also for other bioparticles such as cells, bacteria and viruses. In this manuscript, we have compiled some probable methods that can be used to identify DEP responses of protein without the usage of dye or fluorescent probe, which could change the original state of the protein. From the preliminary experiment of capacitance measurement, we can summarise that the electrical quantification method is reliable for identifying DEP responses. Thus, experimental design should be refined to enhance capacitive measurement’s precision. The quantitative method comprising EIS, CV and capacitance measurement relies on the measurement of load weight which indicates the resistance or capacitance on ROI. These alternatives are a solution for conventional protein analysis techniques as it offers to solve the problem such as time consuming, expensive, inaccurate analysis detection and tedious lab procedures. The further explanation discussed on this paper shows that further studies should be explore DEP for further understanding. 

## Figures and Tables

**Table 1:** Diseases associated with albumin.

Diseases	Definition	References
Hypoalbuminemia	Decrease of albumin inside the body	[9,10,11]
Hyperalbuminemia	Excessive albumin inside the body	[12,13]
Albuminuria	Too much albumin found in the urine.	[14,15]

**Table 2:** Conventional protein analyses and their weaknesses.

Protein Analysis Method	Weakness	References
Enzyme-linked immunosorbent assay (ELISA)	Tedious procedure and the antibody to be prepared is cost-consuming.	[24,25,26,27]
Western blotting	It can only be used if the primary antibody against the specific protein is available. The antibody also can be expensive.	[28,29,30]
Mass spectrometry	Relies on calibration. Miscalibration could lead to an inaccurate result.	[31,32]
Gel electrophoresis	Susceptible to contamination.	[33,34]

**Table 3:** Different types of DEP and their definition.

Types of DEP	Definition	References
Insulator-based dielectrophoresis (iDEP)	An insulating layer or structured insulating arrangement is placed between the electrode where nonuniform fields are being created. Mostly applied with DC with high voltage.	[74,75,76,77]
Electrode-based dielectrophoresis (eDEP)	Nonuniform fields are created by metal-based, sharp-edged with narrow gaps spaced between the electrodes.	[78,79,80]

**Table 4:** Insulator-based DEP type of electrode configuration studied for protein BSA.

Frequency/ Type of Current	DEP Principal Application	DEP Response	Geometry of Electrodes	Conductivity of Suspending Medium	Voltage and Frequency Applied	Reference
DC current	Aggregate trapping	pDEP	Elliptical post array	50 µL phosphate buffer solution; 0.01 S/m	1900 V/cm	[81]
DC current	Enrichment	pDEP	Circular array	Phosphate buffer solution; 0.01 S/m–0.04 S/m	3000 V/cm	[82]
DC current	Enrichment	nDEP	Post array with ‘dove-tail’ geometry on the beginning of the post of both sides.	Deionized water; 25 to 100 S/cm	700 V/cm	[83]
Both AC and DC current	Enrichment	nDEP	Parallel micro ridges	Phosphate buffered saline (PBS); 0.08–0.1 S/m	5 V to 15 V; 10 Hz to 100 kHz	[84]

**Table 5:** Electrode-based-DEP type of electrode configuration studied for protein BSA.

Frequency/ Type of Current	DEP Principal Application	DEP Response	Geometry of Electrodes	Conductivity of Suspending Medium	Voltage and Frequency Applied	Reference
AC current	Trapping	pDEP	Nanohole array	Water medium; 0.28 mS/m	6 Vpp; 1 kHz	[85]
AC current	Enrichment	pDEP	Subarray	Ultrapure water; less than 1 S/cm	0.7–14.1 Vrms; 10 kHz	[86]
AC current	Trapping	pDEP	Column-shaped nanopillar with a flat top	De-ionized water; 2 µS/cm	From 3 MV/m to 5 MV/m	[87]
AC current	Trapping	pDEP	Quadrupole	De-ionized water; 1 mS/m	Up to 8Vpp; 50 kHz and 5 MHz	[88]
AC current	Accumulation	pDEP	Nanocones	Water; $10^{-6}$ S/m	10 V, 2.5 MHz	[89]

**Table 6:** Electrical quantification techniques concept of principles and further explanation.

Electrical Quantification Techniques	Explanation	References
EIS	Defined as the reaction relationship between a biological body and electrical impetus (e.g., extrinsic electrical field) with taking the dielectric properties and frequency applied into account of the reaction	[97]
CV	Described as an electrolytic cell in which three electrodes are dipped into an electrolytic cell, and the measurement of the electric current flow through the cell system.	[106]
Capacitance Measurement	The measurement of changes in the dielectric properties (includes the conductivity and permittivity of the biosample and suspending medium) and the dielectric layer of the sample and suspending medium with the electrode interaction.	[107]

**Table 7:** Correlation between DEP responses with CV.

DEP Responses	Definition	Correlation between DEP and CV
pDEP	Load can be seen attracted on the edge of the electrodes	The adsorption of the sample onto the CV electrodes increases. The peak current of the voltammogram also increases.
nDEP	Load can be seen accumulated in the middle of the electrodes	The adsorption of the sample onto the CV electrodes decreases. The peak current of the voltammogram also decreases.

## Data Availability

The data presented in this study are available within this article.
